# Offering Patients Therapy Options in Unplanned Start (OPTiONS): Implementation of an educational program is feasible and effective

**DOI:** 10.1186/s12882-016-0419-z

**Published:** 2017-01-13

**Authors:** Anna Machowska, Mark Dominik Alscher, Satyanarayana Reddy Vanga, Michael Koch, Michael Aarup, Abdul Rashid Qureshi, Bengt Lindholm, Peter Rutherford

**Affiliations:** 1 Division of Renal Medicine and Baxter Novum, Department of Clinical Science, Intervention and Technology, Karolinska Institutet, M99, Karolinska University Hospital Huddinge, 141 86, Stockholm, Sweden; 2 Robert-Bosch-Krankenhaus, Stuttgart, Germany; 3 University Hospital of North Staffs, Stoke, UK; 4 Nephrologisches Zentrum, Mettmann, Germany; 5 Odense University Hospital, Odense, Denmark; 6 Quintiles, Reading, UK

**Keywords:** Unplanned start, Acute dialysis, Education

## Abstract

**Background:**

Patients with unplanned dialysis start (UPS) have worse clinical outcomes than non-UPS patients, and receive peritoneal dialysis (PD) less frequently. In the OPTiONS study of UPS patients, an educational programme (UPS-EP) aiming at improving care of UPS patients by facilitating care pathways and enabling informed choice of dialysis modality was implemented. We here report on impact of UPS-EP on modality choice and clinical outcomes in UPS patients.

**Methods:**

This non-interventional, prospective, multi-center, observational study included 270 UPS patients from 26 centers in 6 European countries (Austria, Germany, Denmark, France, United Kingdom and Sweden) who prior to inclusion presented acutely, or were being followed by nephrologists but required urgent dialysis commencement by an acutely placed CVC or PD catheter. Effects of UPS-EP on choice and final decision of dialysis therapy and outcomes within 12 months of follow up were analysed.

**Results:**

Among 270 UPS patients who had an unplanned start to dialysis, 214 were able to receive and 203 complete UPS-EP while 56 patients - who were older (*p* = 0.01) and had higher Charlson comorbidity index (CCI; *p* < 0.01) - did not receive UPS-EP. Among 177 patients who chose dialysis modality after UPS-EP, 103 (58%) chose PD (but only 86% of them received PD) and 74 (42%) chose HD (95% received HD). Logistic regression analysis showed that diabetes 1.88 (1.05 – 3.37) and receiving UPS-EP, OR = 4.74 (CI, 2.05 – 10.98) predicted receipt of PD. Patients choosing PD had higher CCI (*p* = 0.01), higher prevalence of congestive heart failure (*p* < 0.01) and myocardial infarction (*p* = 0.02), and were more likely in-patients (*p* = 0.02) or referred from primary care (*p* = 0.02). One year survival did not differ significantly between PD and HD patients. Peritonitis and bacteraemia rates were better than international guideline standards.

**Conclusions:**

UPS-EP predicted patient use of PD but 14% of those choosing PD after UPS-EP still did not receive the modality they preferred. Patient survival in patients choosing and/or receiving PD was similar to HD despite age and comorbidity disadvantages of the PD groups.

**Electronic supplementary material:**

The online version of this article (doi:10.1186/s12882-016-0419-z) contains supplementary material, which is available to authorized users.

## Background

Dialysis initiation through planned fashion with permanent access and a careful organised preparation for the chosen dialysis modality after education is viewed as the most beneficial for patients’ clinical outcomes. However, there is still a significant number of patients that due to unforeseeable deterioration of renal function, delayed presentation to healthcare professionals or other clinical and non-clinical factors, start dialysis in an unplanned manner [1]. This is still a common and important problem in dialysis centres globally and 24-49% of patients are reported to commence dialysis in such a way [1]. According to United States Renal Data System (USRDS) data*,* around 60% of patients who progressed to end-stage renal disease (ESRD) did not have a distinct plan for treatment at the time of start dialysis therapy [2]. Unplanned patients will tend to obtain in-centre HD as a default dialysis option [3], reflected in high usage (up to 80%) of central venous catheter (CVC) [2]. In Europe there is a challenging trend of decreasing use of arteriovenous fistula, AVF (42% in 2005 and 32% in 2009), while CVC use increased from 58% to 68% [4].

The definition of unplanned start (UPS) varies which can make comparisons difficult but in most studies unplanned dialysis start is defined using, in part, first dialysis access with no functional AV fistula or permanent PD catheter. Recently the term “suboptimal” dialysis was proposed to define dialysis commenced as a hospital in-patient, and/or with CVC (without permanent access) [5]. Other criteria for defining UPS have also been proposed:

1. Late referral defined as time between referral to the nephrology unit and first dialysis ranging between 1 and 6 months [6]. Late referral is not entirely synonymous with UPS; however, early referral tends to be a predictor of better coordination of medical care in pre-dialysis stage, management of CKD complications, and education around dialysis option that is based on informed consent, and may therefore decrease probability of UPS. A recent meta-analysis shows that early referral is associated with reduced mortality and hospitalization, greater uptake of PD and timely placement of permanent dialysis access [7]. This is clinically important as patients who start dialysis with CVCs have increased chances of prolonged CVC use and associated complications [8].
2. Biochemical parameters e.g. estimated glomerular filtration rate (eGFR) - defined as early (above a certain level of eGFR) or late (below that level of eGFR) start which can be misleading as it does not reflect a clinical pathway. The randomized, multicentre, controlled IDEAL study aimed to evaluate the optimal dialysis start based on estimated GFR (eGFR) [9] of early vs late initiation. There was no difference in terms of survival between these eGFR defined groups but more patients in the “late” start category had UPS with temporary access.
3. Speed of the need for dialysis - emergent dialysis, urgent dialysis and non-urgent dialysis as defined by Ghaffari [3]: Emergent start < 48 hours, urgent start > 48 hours and up to 2 weeks, whilst non-urgent start were those that were able to plan and start with their modality of choice [3].
4. Being known or “unknown” to nephrology care. There are “known” patients that despite nephrology follow up, have UPS due to unpredictable GFR decline or care pathway failures. In addition, there is a cohort of truly “unknown” patients that present with undiagnosed CKD stage 5.

Despite discrepancies in this clinical nomenclature which makes comparisons challenging, it is clear that UPS patients have more clinical problems such as increased morbidity and mortality [10], increased use of healthcare resources (e.g. hospital days) [11] and are less likely to receive a choice of dialysis modality and choose a home dialysis therapy, and typically start on in-centre HD, compared with patients starting planned dialysis [12]. This is partly access driven as patients starting with a CVC have higher mortality risk as compared with those using PD or start HD with AVF or arteriovenous grafts [13] and have increased risk of septicaemia [14]. Studies evaluating whether it is possible to educate UPS patients and commence or switch early to PD therapy are relatively infrequent; however, single centre studies show that UPS patients can commence PD [3, 15–18] and PD in UPS patients can give outcomes similar to unplanned HD [19, 20].

However, clinical concerns remain over UPS and whether it is even feasible to educate UPS patients who generally have started on dialysis around different modalities and whether the system of care in dialysis units can be organized to educate and deliver choice of dialysis modality. Therefore this study was designed to examine the feasibility and impact of an educational programme intended to affect the UPS patient pathway (UPS-EP) and deliver a tailored educational programme to allow modality choice.

## Methods

### Study design

This was a non-interventional, prospective, multi-centre, observational study of unplanned start (UPS) patients, who all received dialysis therapy, with up to 12 months follow up time. Participating centres had implemented the UPS-EP into their routine clinical practice. The development and implementation of the UPS-EP has been described elsewhere [21] but briefly consists of;

1. Analysis of UPS patient flow in a dialysis unit with process improvement approach to understand and resolve issues - the units mapped out their unplanned start pathway to understand and improve specific bottlenecks and constraints. The aim was to improve the pathway to patient education, decision making and formation of permanent access (AV fistula or catheter).
2. A specific patient education program focusing on the dialysis modality choice facing UPS patients, who had started on dialysis in an unplanned fashion, supported by decision support tools. This was developed in collaboration with 5 dialysis units in Europe and academic institutions specialized in patient education and aims at facilitate the decision making process for dialysis modality choice. There is no global consensus regarding the structure and content for modality education although standards have been suggested for planned start CKD education programs [22]. Programs tend to focus on general CKD knowledge, treatment as well as dialysis modality decisions [23]. In contrast, the UPS-EP educational focus is on the modality decision itself since this is the critical element of education to improve health literacy at UPS. This approach is followed in other conditions eg oncology [24, 25] since at diagnosis, patients need treatment option information, and to understand the impact of the disease and the treatment options on themselves. Thus, the UPS-EP included information on HD, PD, home HD and conservative care as well as transplantation and was delivered to the patients during at least 3 individual sessions by nurses using motivational interviewing methodology, at the pace determined by the nurse assessing the clinical condition. Educational material included a dialysis options booklet matching the educational material delivered by the nurse, a photograph based book showing HD, PD and home HD and a unit-specific modality video alongside HD unit visit and demonstration of PD. In addition, the decision support tools within the UPS-EP contained three aids, chosen by the educators for individual patients from the Ottawa online decision aid, a self-completion balance scale, and a set of decision cards which allowed the patient to prioritize the value to them of specific issues and factors which related to CKD treatment.

Twenty-six centres in 6 European countries (Austria, Denmark, Germany, France, Sweden and United Kingdom) aimed to recruit all UPS patients presenting in their units. In these centres, all UPS patients were identified on clinical presentation and considered actively for education within the structured UPS-EP with the use of decision support tools. UPS patients who were judged clinically to not be suitable for this educational approach or would not be able to make a modality choice for medical reasons were still identified and included in the overall UPS cohort. Patients could receive the UPS-EP at the time of presentation or following dialysis start.

### Inclusion and exclusion criteria

As discussed in the introduction the literature around patient definition is confusing but this study aimed to focus on patients based on the nature of the dialysis start rather than simply on the referral timing or the speed of first dialysis start following access placement. UPS patients were considered eligible for the study on the basis of the following inclusion criteria;

1. they had CKD stage 5,
2. were aged between 18 and 90 years old at the time when informed consent was signed, and
3. had commenced dialysis in an unplanned way on the basis of clinical criteria of presentation to the nephrologist within 1 month of needing dialysis (as ‘unknown” patient) AND/OR being followed by nephrologist but requiring urgent dialysis commencement by CVC or an acutely placed PD catheter.

The exclusion criteria included a diagnosis of acute kidney injury (AKI) rather than CKD stage 5 - given in NICE Guideline [26] - a clinical decision to actively follow a conservative clinical management plan (chronic dialysis is not to be performed) and other serious or acute conditions that, in the investigator’s opinion, would preclude participation in the study or where life expectancy was estimated at 6 months or less.

Patients gave informed consent for inclusion and data collection at the time of UPS presentation or in the recovery phase around the time of hospital discharge. The scheme of the study recruitment and follow up is presented as Additional file 1: Figure S1 in additional file.

### Data collection

Demographic or clinical data were collected either from the patients’ healthcare records or from routine patient-health care professional interactions at baseline, 6 months and 12 months. This included patient demographics, medical history including comorbidities assessed by Charlson comorbidity index (CCI), dialysis access procedures, details of the presentation with end-stage renal failure requiring UPS and details of starting modality, access interventions and the number and length of hospitalization linked to UPS. Data collection at 6 months (defined as 6 months following first HD session or first PD day at home if PD from the start of dialysis) and 12 months (defined as 12 months following first HD session or first PD day at home if PD from the start of dialysis) recorded patient status, dialysis modality, and if, when changed, details of dialysis access procedures, brief details of dialysis related infectious events and number and length of any hospitalizations.

### Impact of UPS-EP and patients flow

The primary objective of the study was to evaluate the impact and effectiveness of the UPS-EP on the choice of dialysis therapy (HD or PD) made by UPS patients who had been initiated on dialysis in an unplanned fashion. In addition, the patient flow through the educational program to ultimate decision making was analysed by logistic regression to identify predictors of receiving PD vs HD dialysis therapy at any time point during the study, including PD as an initial UPS dialysis modality. The dependent categorical variable was PD or HD. Explanatory variables included in the model were: age, gender, eGFR at first dialysis session, presence of diabetes, hospitalization for UPS, time between first referral to nephrologist and first dialysis session and received education. The flow of patients into and through UPS-EP in terms of eligibility and feasibility of education, completion of education program and decision making was collected.

### Peritonitis and bacteraemia rate

Peritonitis rate was calculated according to ISPD guidelines [27] and expressed as months of PD at risk, divided by number of episodes, and expressed as interval in months between episodes and also as number of infections for a time period, divided by dialysis-years’ time at risk, and expressed as episodes per year.

In order to compare the severity of the infection events we applied the same calculation scheme to show the bacteraemia rate among HD patients calculated based on the number of haemodialysis bloodstream infection. In our study, the definitions are classified according to the KDIGO Vascular Access guidelines [28]. Bloodstream infection was defined as blood culture results positive for the presence of bacteria with or without the accompanying symptom or fever.

During the study, one patient undergoing HD had a peritonitis event, this patient was initially managed with HD, chose PD but peritonitis occurred during the surgical procedure of PD catheter placement, and PD was never performed. For the purpose of this analysis of peritonitis rate during PD therapy in UPS patients, this peritonitis event was not taken into account.

### Statistical methods

Data are expressed as median (10th to 90th percentile) or percentage or odds ratio (95% CI, confidence intervals), as appropriate. Statistical significance was set at the level of *p* < 0.05. For comparison between two groups non –parametric Wilcoxon test was used - and for three or more groups - non-parametric Kruskal-Wallis ANOVA test was used. Chi-square test was used for nominal variables. Logistic regression was performed to see the predictors of receiving PD vs HD dialysis therapy during the study, including initial dialysis modality. Explanatory variables in the model include age, gender, eGFR, presence of diabetes, hospitalization for unplanned start, time between first referral to nephrologists and first dialysis session and received education. Kaplan Meier survival analysis was performed to investigate one-year survival. We did not take under consideration multiple comparisons; therefore the presented data have a descriptive nature. Statistical analyses were performed using statistical software SAS version 9.4 (SAS Campus Drive, Cary, NC, USA).

## Results

### Baseline characteristics of UPS patients

Two hundred and seventy UPS patients who had an unplanned dialysis start were recruited in the OPTiONS Registry, 230 were followed up for 6 months and 197 patients completed 12 months follow-up. Seventy three patients were prematurely withdrawn from the study due to death (*n* = 47), renal transplantation (*n* = 10), lost to follow-up (*n* = 7) or other reason (*n* = 8), whereas only one patient voluntary withdraw from the study. Fourteen patients died during the first 90 days after commencing dialysis. The diagram showing the flow of 270 UPS patients enrolled in the study is presented in additional file as Additional file 1: Figure S2

The baseline characteristics of UPS patients are presented in Table 1. Their median age was 69 years, and 64% were males. At inclusion, comorbidities included diabetes (41%), congestive heart failure (31%), myocardial infarction (18%) and peripheral vascular disease (18%), and the median value of CCI was 6. Patients were referred to the nephrology unit equally often from primary care and from other hospital specialties, and the majority were referred during an in-patient admission. The majority of patients were hospitalized for the UPS (91%). The median eGFR level at the time of first dialysis initiation was 7 ml/min/1.73 m^2^. Only 17% of patients initiated dialysis on the referral day, 32% patients received their first dialysis after the first day but within 2 weeks, 15% had their first dialysis 2 weeks to 3 months, and 36% after more than 3 months after presenting.

**Table 1:** Baseline characteristics of all 270 UPS patients, 214 patients who received UPS-EP and 56 patients who did not receive UPS-EP

	UPS patients n = 270	Received UPS-EP *n* = 214	Not received UPS-EP *n* = 56	*P* value
Age	69 (40-83)	67 (37-84)	72 (54 – 83)	**0.01**
Sex, M/F, %	64/36	64/36	64/36	1.00
eGFR, ml/min /1.73 m^2^ BSA	7 [4–16]	7 [4–16]	7 [4–16]	0.53
Charlson comorbidity index, CCI	6 [2–10]	6 [2–10]	7 [5–10]	**<0.01**
Comorbidities, %				
* Diabetes* * Congestive heart failure* * Myocardial infarct* * Peripheral vascular disease*	41 31 18 18	39 28 17 16	50 41 21 25	0.17 0.07 0.56 0.12
Primary renal disease^a^, %				
* Chronic renal failure, etiology unknown* * Glomerulonephritis* * Renal vascular disease* * Diabetic nephropathy* * Other* ^*b*^	13 20 20 25 22	12 22 18 23 25	14 14 16 32 24	0.59
Patients source, %				
* In-patient admission* * Out-patient referral*	71 29	68 32	80 20	0.09
Referral, %				
* Primary care* * Other hospital specialty* * Missing/unknown*	51 48 1	53 46 1	48 52 0	0.64
Hospitalization for the unplanned start, %	91	90	93	0.80
Countries, %				
* United Kingdom* * Germany* * Denmark* * Sweden* * Austria* * France*	24 36 18 4 11 7	29 34 17 5 7 8	5 43 20 2 28 2	**<0.001**

Values are expressed as median (10-90 percentiles) or percentage. CCI, Charlson comorbidity index; eGFR, estimated glomerular filtration rate, significant values are marked in bold (*p* < 0.05)

^a^Cause of renal disease

^b^Pyelonephritis, interstitial nephropathy, cystic kidney disease, inherited renal disease, renal hypoplasia, multisystem renal disease, myeloma, amyloid, other renal disease

**Fig. 1:**
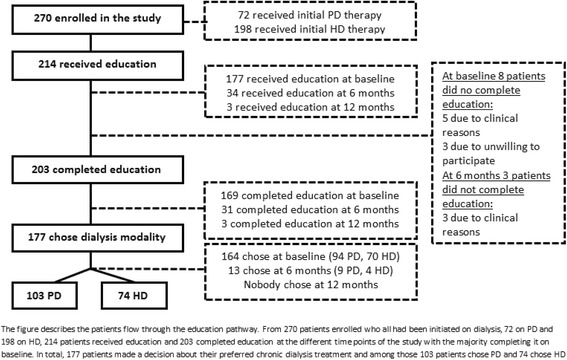
Flow of 270 UPS patients through UPS-EP

The majority of UPS patients were able to receive UPS-EP (*n* = 214) whereas 56 patients never received education (Fig. 1). Some (*n* = 104) patients were reported by the clinical teams as not being suitable as a PD candidate and reasons other than patient choice (22%) were as follows: obesity (2%), hernia (1%), previous surgical scarring/adhesions (6%), domestic circumstances (4%), cognitive barriers (5%) and physical barriers (6%).

Patients who never received UPS-EP were significantly older (*p* = 0.01), more comorbid (CCI, *p* < 0.01), and they were distributed differently across the countries (Table 1). There was a trend regarding patient source with a higher in-patient admission in the group who never received UPS- EP (*p* = 0.09).

### Effect of UPS-EP

The majority, 214 out of 270 patients who had an unplanned dialysis start, were suitable for making decision on preferred dialysis modality after receiving and completing UPS-EP (Fig. 1). Most of the 177 patients (177/214) received UPS-EP immediately or shortly after UPS presentation, 34 patients within the first 6 months after commencing dialysis, and 3 patients after 12 months. Whereas 203 out of the 214 UPS-EP patients completed the educational programme, 11 patients did not complete because of their medical condition (*n* = 8) or unwillingness to participate (*n* = 3). Following the completion of UPS-EP, 177 patients made a decision on initial dialysis modality, 103 patients chose PD and 74 HD, while 26 patients did not make, or were unable for clinical reasons to make a decision on a preferred dialysis therapy, following completion of UPS-EP.

Logistic regression analysis of factors influencing the dialysis modality received among the 270 UPS patients enrolled in the study showed that having diabetes: OR = 1.88 (CI, 1.05 – 3.37) and receiving UPS-EP: OR = 4.74 (CI, 2.05 – 10.98) were statistically significant predictors of receiving PD (Table 2). Including CCI as a variable in the model had no additive value and was not a significant predictor of receiving PD (data not shown).

**Table 2:** Logistic regression analysis evaluating predictors of receiving PD therapy in 270 UPS patients

Pseudo r = 0.09	Odds ratio (95% CI)	*P* value
Age, ≥ 69 years	1.26 (0.71 – 2.24)	0.42
Gender, male versus female	1.51 (0.82 - 2.78)	0.19
eGFR, ≤7 ml/min/1.73 m^2^	1.33 (0.75 – 2.35)	0.33
Diabetes, presence versus absence	**1.88 (1.05 – 3.37)**	**0.03**
Hospitalization, yes/no	0.51 (0.21 – 1.30)	0.16
Time between first referral to nephrologists and first dialysis, ≥ 15 days	0.84 (0.48 – 1.48)	0.55
UPS-EP, received versus not received	**4.74 (2.05 – 10.98)**	**<0.001**

*CCI* Charlson comorbidity index, *eGFR* estimated glomerular filtration rate, significant values are marked in bold (*p* < 0.05)

Median age: 69 years; median eGFR: 7 ml/min/1.73 m2; median time between first referral to nephrologists and first dialysis: 15 days;

### Characteristics of patients completing UPS-EP and making modality choice

We looked for underlying demographic differences in the characteristics of UPS patients who completed UPS-EP and declared a choice of PD or HD since differences could then influence clinical outcomes observed over follow up. Table 3 shows the clinical characteristics of the 177 patients who completed the UPS educational program and chose a preferred dialysis modality, PD (*n* = 103) or HD (*n* = 74). These two groups could be considered as analogous to the “intention to treat” populations in studies comparing two different therapies - the PD group and HD group are defined by their preferred choice of modality, not actual modality. Patients who chose PD were more comorbid (CCI, *p* = 0.01), with higher prevalence of congestive heart failure (*p* < 0.01), were distributed differently across countries (*p* < 0.01) and with the trend towards higher number of in-patient admissions (*p* = 0.05) than in the HD choosing group. These results demonstrate that PD was not being selected only by younger and fitter patients and that a wide range of patients were receiving education and choosing PD. There were 7 patients whose first modality was HD who all completed USP-EP decided to remain on HD and not switch to PD but expressed a choice of transfer to Home HD. It is unclear how many were trained and transferred during the study but all remained on HD so are included within the HD group.

**Table 3:** Clinical characteristics of 177 patients who completed UPS-EP and chose a preferred dialysis modality: PD or HD

UPS patients who completed education and chose dialysis modality (*n* = 177)		PD (n = 103)	HD (*n* = 74)	*P* value
Age, years	67 (38-85)	71 (36-86)	64 (4-82)	0.09
Sex, M/F, %	69 / 31	66 / 34	73 / 27	0.41
eGFR, ml/min /1.73 m^2^ BSA	7 [4–16]	7 [4–19]	7 [3–12]	0.34
Charlson comorbidity index, CCI	6 [2–10]	7 [2–10]	5 [3–9]	**0.01**
Comorbidities, %				
* Diabetes* * Congestive heart failure* * Myocardial infarct* * Peripheral vascular disease*	41 29 18 14	45 39 22 17	35 16 11 9	0.22 **<0.01** 0.07 0.19
Primary renal disease^a^, %				
* Chronic renal failure, etiology unknown* * Glomerulonephritis* * Renal vascular disease* * Diabetic nephropathy* Other^b^	12 20 18 23 27	12 28 17 23 20	12 17 20 22 29	0.32
Patients source, %				
* In-patient admission* * Out-patient referral*	68 32	74 26	59 41	0.05
Referral, %				
* Primary care* * Other hospital specialty* * Missing/unknown*	59 40 1	65 34 1	51 49 0	0.09
Hospitalization for the unplanned start, %	89	87	91	0.51
Countries, %				
* United Kingdom* * Germany* * Denmark* * Sweden* * Austria* * France*	33 40 10 6 3 8	28 48 13 7 1 3	41 27 7 5 5 15	**<0.01**

Values are expressed as median (10-90 percentiles) or percentage. CCI, Charlson comorbidity index; eGFR, estimated glomerular filtration rate. Significant differences are marked in bold (*p* < 0.05)

^a^Cause of renal disease

^b^Pyelonephritis, interstitial nephropathy, cystic kidney disease, inherited renal disease, renal hypoplasia, multisystem renal disease, myeloma, amyloid, other renal disease

### Characteristics of patients who did or did not receive their preferred dialysis modality

Among the investigated UPS patients not all received the modality that they chose after education and supported decision making. We compared PD and HD patients who at any point of the study received their chosen dialysis modality (Additional file 1: Table S1). In our study, 89 patients chose and received PD and 70 patients chose and received HD according to their recorded decision. PD patients were significantly more comorbid (CCI, *p* = 0.02), had more often medical history of congestive heart failure (*p* < 0.001) and myocardial infarction (*p* = 0.02), were significantly more often from in-patient UPS admission (*p* = 0.02) or referred form primary care (*p* = 0.04), and were differently distributed among participating countries (*p* < 0.001).

In addition, we characterized patients who throughout the study never received their expressed will of choice of dialysis modality: 14 patients chose and never received PD, and 4 patients chose but never received HD. On comparing those groups with the groups that received their chosen modalities to investigate putative difference in patient characteristics there were no significant differences between those groups (Additional file 1: Table S1). In particular patients who chose and received PD were not different clinically from those who chose but did not receive PD.

### Characteristics of patients who actually received PD and HD

Since not all the patients received their preferred therapy we compared patients who completed the UPS-EP according to the modality that they actually received. These groups are analogous to the “as treated” groups in a clinical trial of two therapies. The group treated by PD contains patients who did receive their PD choice (*n* = 89) and patients who chose HD and never received it (i.e., did not switch from PD to HD), and therefore were assigned to PD (*n* = 4) by the treating clinical team for clinical reasons. Treated by HD group contains patients who received their HD choice (*n* = 70) and patients who chose PD but never received it (i.e., did not switch from HD to PD), and therefore were assigned to HD (*n* = 14) by the clinical team. The characteristics of the groups are presented in Table 4. The results show that compared to treated by HD patients, treated by PD patients were significantly more comorbid (CCI, *p* = 0.04), with higher prevalence of congestive heart failure (*p* < 0.01) and, were significantly more often from in-patient admission (*p* = 0.02), and referred from primary care (*p* = 0.02), and were differently distributed among participating countries (*p* < 0.001).

**Table 4:** Clinical characteristics of 177 patients completing UPS-EP according to their actual received modality, PD (n = 85) or HD (n = 92)

	Received PD (n = 93)	Received HD (n = 84)	*P* value
Age, years	70 (36-86)	65 (41-82)	0.37
Sex, M/F, %	68/32	70/30	0.75
eGFR, ml/min /1.73 m^2^ BSA	7 [4–18]	7 [4–15]	0.97
Charlson comorbidity index, CCI	7 [2–10]	6 [3–9]	**0.04**
Comorbidities, %			
* Diabetes* * Congestive heart failure* * Myocardial infarction* * Peripheral vascular disease*	47 40 23 16	33 18 12 11	0.07 **<0.01** 0.08 0.38
Primary renal disease^a^, %			
* Chronic renal failure, etiology unknown* * Glomerulonephritis* * Renal vascular disease* * Diabetic nephropathy* Other^b^	12 29 15 25 19	12 17 21 20 30	0.18
Patients source, %			
* In-patient admission* * Out-patient referral*	76 24	58 42	**0.02**
Referral, %			
* Primary care* * Other hospital specialty* * Missing/unknown*	68 31 1	50 50 0	**0.02**
Hospitalization for the unplanned start, %	88	89	1.0
Countries, %			
* United Kingdom* * Germany* * Denmark* * Sweden* * Austria* * France*	25 54 11 7 1 2	43 24 9 5 5 14	**<0.001**

Values are expressed as median (10-90 percentiles) or percentage.

CCI, Charlson comorbidity index; eGFR, estimated glomerular filtration rate

^a^Cause of renal disease

^b^Pyelonephritis, interstitial nephropathy, cystic kidney disease, inherited renal disease, renal hypoplasia, multisystem renal disease, myeloma, amyloid, other renal disease

### Clinical outcomes in UPS patients

We investigated clinical outcomes of patients who chose and received PD (*n* = 89) and chose and received HD (*n* = 70) to assess clinical outcomes in terms of survival and infection rates.

Using Kaplan-Meier analysis we found no significant difference in 1-year survival between patients who chose and received PD and HD. We compared PD and HD with the addition of the group of patients who chose and never received PD (*n* = 14) to the HD group. There was no significant difference in 1-year survival between the PD and HD group. Finally, there was no significant difference in 1-year survival between patients who were actually treated with PD (*n* = 93) and HD (*n* = 84) respectively.

The overall peritonitis rates in PD patients were lower than ISPD recommended targets [27]: Peritonitis rate in PD (*n* = 89) patients who chose and received PD was 1 episode per 58.1 patient months (0.21 episodes per year) and peritonitis rate in all PD (*n* = 93) patients was 1 episode per 60.2 patient months (0.20 episodes per year). Similarly, the bacteraemia rates were as follows in the HD patients: bacteraemia rate in HD (*n* = 70) patients who chose and received HD was 1 episode per 76.8 patient months (0.16 episodes per year) and bacteraemia rate in all HD (*n* = 84) patients was 1 episode per 66.9 patient months (0.18 episodes per year).

## Discussion

UPS patients represent a challenge in every dialysis unit and have poor clinical outcomes – partly due to morbidity and mortality associated with CVC use – compared to planned start patients [10, 11, 29, 30]. The challenges facing UPS patients were explored in the present study which provided answers to several important questions;

1. What are the characteristics of a current cohort of UPS patients and are there issues around patient management which are amenable to change?
2. Is it possible to deliver an educational program to UPS patients to allow dialysis choice?
3. What are the outcomes if that choice is put into practice for UPS patients?

The OPTiONS study shows that UPS patients are similar in terms of demographics to the incident dialysis population in the same European countries in regards to age and gender distribution [31] with the only difference being a higher prevalence of diabetes in the OPTiONS study. Thus there are no fundamental case mix differences in UPS patients which should affect their ability to receive education and make choices. However, there is no additional information regarding other factors e.g. inflammatory biomarkers [32] which are associated with comorbid illness and CKD and are associated with survival differences in patients starting dialysis. Of course, the timing of UPS-EP needs to consider the general condition and receptivity of the patient at the time of UPS. Moreover, at dialysis start, our patients had eGFR level comparable with other studies [33, 34] which confirms that firstly, UPS should not be defined by a particular eGFR level, often an element of confusion over early vs late start. Secondly, the eGFR levels, although variable, do not suggest that the majority of patients are progressing rapidly or are presenting with minimal residual renal function (RRF) and previously unrecognized CKD. This is confirmed by our study since only a relatively small percentage of patients required dialysis therapy on the day of referral to the nephrology unit. In the majority of patients there was an interval of several days – allowing some time for preparation and education. There is still a significant minority of UPS patients who have been first referred many months prior to first dialysis. This study cannot confirm whether or not these patients received ongoing nephrological care in a specialist clinic but points to the observation that failures of the care pathway in terms of patient follow up or preparation or unexpected changes in the RRF decline trajectory are likely causative factors of an unplanned start in this group of patients. Further work would be needed to examine this in more detail and develop pathway support tools or more accurate predictive models/monitoring approaches to prevent UPS.

The benefits of patient dialysis modality education are well known [24] but it could be perceived that UPS patients are too unwell/unstable to receive education and make clinical decisions. However, more recent studies [3, 15, 16], albeit with a range of different UPS definitions and inclusion criteria and often single centres, have shown that education is possible and dialysis choice can be facilitated and increase the possibility of patients to receive home based therapy [35]. The OPTiONS study confirms this is possible, in a range of different renal units across different European healthcare systems, confirming that it is achievable to a similar degree to that reported in specific dialysis centres with particular practice patterns [16, 19, 20]. The wide inclusion criteria of this study was designed to allow most UPS to be included but even so most patients were able to commence (79%) and complete education (75%). This study employed decision support tools for the first time in UPS education, which in other long term conditions have been shown to facilitate decision making [36] by patients. This helped the majority of patients completing the education to make a decision and communicate it to their renal team. Receiving UPS-EP was a highly significant predictor for receiving PD in the logistic regression model. There are only small differences in patient demographics between patients who did and did not receive UPS-EP so there should be careful clinical assessment before making judgements over suitability for education in UPS patients to avoid unwarranted exclusion. A small minority still received UPS-EP up to 6 months. Therefore design of an UPS programme should allow an approach to “capture” or “revisit” patients following their UPS to ensure equity of access to dialysis modality choice. The choice of modality, PD or HD, was also interesting with perhaps counterintuitively PD choosing patients being older and more comorbid compared to those choosing HD. In part this may reflect the practice patterns of some of the units involved in OPTiONS with experienced PD practitioners who may have advocate practice patterns supportive of PD for patients with for example CHF [20]. This is a factor within the country effect noted in the study reflecting underlying differences in practices which play a role in modality choice. Diabetic patients were more likely to receive PD although there is no clear evidence of benefit of PD or HD in diabetic patients [37] and recent ERA-EDTA data [38] show identical proportions of diabetic patients in PD and HD in Europe. OPTiONS has also uncovered that education and decision making are still not enough in terms of delivering modality choice – a small but significant number of patients expressed a preferred choice but did not receive their chosen modality. This was particularly seen in patients who chose PD but remained on HD. This study could not determine the precise reasons but it does not appear to relate to patients clinical characteristics e.g. age, comorbidity etc. Other factors could include inability to form PD access (although only seen in one patient in this study), an “overruling” judgement from the physician, a change of the decision by the patient, or other healthcare process issues. One solution could be a case manager [23] to follow patients and navigate the care pathway once a decision is made to ensure that decisions are enacted wherever clinically possible. Ensuring effectiveness of the access care pathway is also important, 72 UPS patients commenced dialysis with PD without a need of temporary HD through a combination of medical and surgical catheter insertion programs and PD start in a more urgent way.

Although patient centric care should support shared decision making after patient education [39], it is important to assess whether the choices made are “good ones” by examining clinical outcomes. Overall patient survival was the same in patients choosing and/or receiving PD compared to those treated by HD despite the age and comorbidity disadvantages of the PD groups. Perhaps more importantly, in the PD patients, the peritonitis rate was well below the recommended ISPD target and better than other European contemporary data [40]. OPTiONS was not powered to measure survival as a primary outcome measure and only 1 year follow up was performed, nevertheless there are no indications that patient choice of modality associated with poor clinical outcomes in UPS patients.

Our study has several strengths. This was multicentre study, so our findings could be applicable to other dialysis centres elsewhere (high external validity). We aimed to analyse a contemporary cohort of UPS patients including as many cases of unplanned dialysis start in the centres as possible. Moreover we have conducted the study in real clinical settings and investigated the treatment pathways and challenges related to UPS education, switching dialysis modality, and hospitalization within a complex study population. However, several limitations should be highlighted; it was a non-interventional, observational study, therefore a random group allocation was not performed and confounding factors are likely to play an important role. A randomized controlled trial of education could in theory be performed in a cluster randomized approach but this would be complicated by ethical concerns in denying modality choice when this is known to be important in determining clinical outcomes. Furthermore no control group to rigorously measure the dialysis choices and outcomes made in the same or different centres over a different time period was included; however, it is described consistently that renal units without a defined UPS programme have low use of PD. The 12 months follow-up period allowed us to only speculate on long term clinical outcomes of patients. Since this was a “real world” study, clinical outcomes were limited with no measurement of parameters such as blood pressure, fluid status, dialysis adequacy or other biochemical/haematological parameters. Finally, we did not study the patients prior to enrolment; thus we could not analyze the specific clinical conditions which had prompted dialysis initiation. Overall, while the focus of OPTiONS was on the impact of an educational programme, its findings and strengths are in our opinion potentially relevant for all nephrology practitioners and could impact on everyday clinical practice.

## Conclusions

The OPTiONS study of the feasibility and effectiveness of implementing the UPS-EP, a program focusing on patient flow and a specific educational programme with decision support, shows that UPS (defined by referral to nephology within a month of start AND/OR acute dialysis with CVC or PD catheter) patients who completed UPS-EP, were more likely to choose PD as their preferred modality. This indicates that a programme such as the UPS-EP makes it possible to organise patient’s pathways in dialysis units to facilitate informed choice. These findings suggest that education facilitating informed choice of home based dialysis therapies among UPS patients may lead to better clinical outcomes and optimization of healthcare resource utilisation although this need to be confirmed in further studies.

## Additional file

Additional file 1: The additional material file contains: **Figure 1s** - Study scheme for inclusion, consent and follow up of UPS patients. **Figure 2s** - UPS flow during study follow up. **Table 1s** - Clinical characteristics of 177 patients who chose and received PD (*n* = 89) or HD (*n* = 70) or who chose but never received PD (*n* = 14) or HD (*n* = 4). (DOCX 89 kb)

## Abbreviations

AKI: Acute kidney injury
AVF: Arteriovenous fistula
AVG: Arteriovenous graft
CCI: Charlson comorbidity index
CHF: Congestive heart failure
CKD: Chronic kidney disease
CVC: Central venous catheter
eGFR: Estimated glomerular filtration rate
ESRD: End stage renal disease
HD: Haemodialysis
PD: Peritoneal dialysis
RRF: Residual renal function
UPS: Unplanned start
UPS-EP: Unplanned start educational programme
USRDS: United States Renal Data System
