# Supplementary material for: Offering Patients Therapy Options in Unplanned Start (OPTiONS): Implementation of an educational program is feasible and effective
Source: BMC Nephrol. 2017 Jan 13;18:18. doi: 10.1186/s12882-016-0419-z (PMC5237347; doi:10.1186/s12882-016-0419-z)
Supplement: Additional file 1: — The additional material file contains: Figure 1s - Study scheme for inclusion, consent and follow up of UPS patients. Figure 2s - UPS flow during study follow up. Table 1s - Clinical characteristics of 177 patients who chose and received PD (n = 89) or HD (n = 70) or who chose but never received PD (n = 14) or HD (n = 4). (DOCX 89 kb) [file 12882_2016_419_MOESM1_ESM.docx]

Figure 1s Study scheme for inclusion, consent and follow up of UPS patients


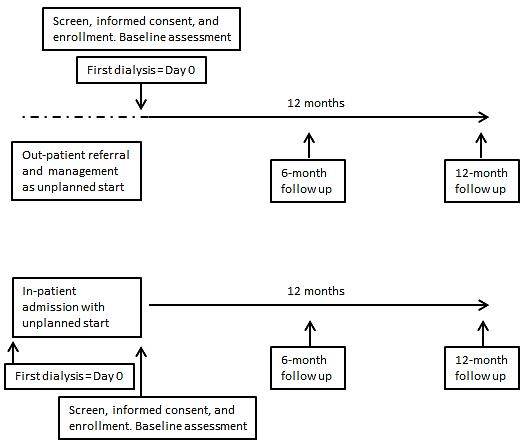


Figure 2s UPS flow during study follow up


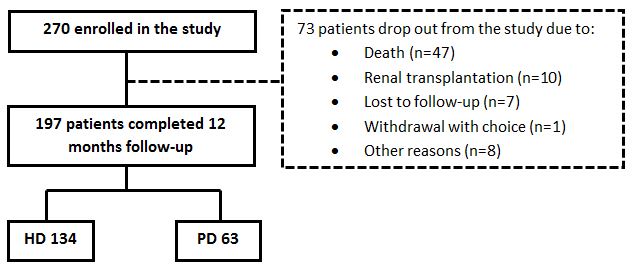


Table 1s Clinical characteristics of 177 patients who chose and received PD (n=89) or HD (n=70) or who chose but never received PD (n=14) or HD (n=4)

|  | **Choose and received PD (n=89)** | **Chose and received HD (n=70)** | **P value* (n=159)** | **Chose and never received PD (n=14)** | **Chose and never received HD (n=4)** | **P value** (n=177)** |
| --- | --- | --- | --- | --- | --- | --- |
| Age, years | 71 (35-87) | 64 (40-82) | 0.17 | 74 (45-86) | 61 (49-82) | 0.31 |
| Sex M/F | 66/34 | 71/29 | 0.61 | 64/36 | 100/0 | 0.31 |
| eGFR, ml/min /1.73 m^2^ BSA | 7 (4-17) | 7 (4-11) | 0.60 | 9 (5-32) | 6 (3-20) | 0.42 |
| Charlson comorbidity index, CCI | 7 (2-10) | 5 (2-8) | **0.02** | 7 (3-10) | 6 (4-10) | 0.09 |
| **Comorbidities**  *Diabetes*  *Congestive heart failure*  *Myocardial infarct*  *Peripheral vascular disease* | 46  40  20  17 | 33  16  7  10 | 0.10  **<0.001**  **0.02**  0.25 | 36  29  36  14 | 75  25  75  0 | 0.17  **<0.01**  **<0.01**  0.43 |
| **Primary renal disease^a^**  *Chronic renal failure, etiology unknown*  *Glomerulonephritis*  *Renal vascular disease*  *Diabetic nephropathy*  Other^b^ | 12  29  16  24  19 | 13  16  21  20  30 | 0.20 | 8  21  21  21  29 | 0  25  0  50  25 | 0.61 |
| **Patients source**  *In-patient admission*  *Out-patient referral* | 75  25 | 57  43 | **0.02** | 64  36 | 100  0 | **0.03** |
| **Referral**  *Primary care*  *Other hospital specialty*  *Missing/unknown* | 66  33  1 | 49  51  0 | **0.04** | 57  43  0 | 100  0  0 | 0.09 |
| **Hospitalization for the unplanned start, %** | 88 | 90 | 0.64 | 86 | 100 | 0.73 |
| **Countries**  *United Kingdom, %*  *Germany, %*  *Denmark, %*  *Sweden , %*  *Austria, %*  *France, %* | 26  52  11  8  1  2 | 43  23  7  6  6  15 | **<0.001** | 43  29  21  0  0  7 | 0  100  0  0  0  0 | **0.001** |

Values are expressed as median (10-90 percentiles) or percentage. CCI, Charlson comorbidity index; eGFR, estimated glomerular filtration rate; * p value, comparison between 2 groups: those who chose and received PD vs chose and received HD; **p value, comparison between all groups

a Grouping cause of renal disease

b Pyelonephritis, interstitial nephropathy, cystic kidney disease, inherited renal disease, renal hypoplasia, multisystem renal disease, myeloma, amyloid, other renal disease.
